# Medical and Chirurgical Society of London

**Published:** 1845-04

**Authors:** 


By some of the recent journals, we learn that Professor Thomas D. Mütter, of Philadelphia, has been elected a member of the Royal Medical and Chirurgical Society of London.

